# Assessing the influence of landscape conservation and protected areas on social wellbeing using random forest machine learning

**DOI:** 10.1038/s41598-024-61924-4

**Published:** 2024-05-18

**Authors:** Joshua Fisher, Summer Allen, Greg Yetman, Linda Pistolesi

**Affiliations:** 1 AC4, Columbia Climate School, Columbia University, New York, NY USA; 2 Network for Education and Research on Peace and Sustainability, Hiroshima University, Higashi-Hiroshima, Japan; 3 CIESIN, Columbia Climate School, Columbia University, New York, NY USA

**Keywords:** Conservation biology, Environmental sciences, Environmental social sciences

## Abstract

The urgency of interconnected social-ecological dilemmas such as rapid biodiversity loss, habitat loss and fragmentation, and the escalating climate crisis have led to increased calls for the protection of ecologically important areas of the planet. Protected areas (PA) are considered critical to address these dilemmas although growing divides in wellbeing can exacerbate conflict around PAs and undermine effectiveness. We investigate the influence of proximity to PAs on wellbeing outcomes. We develop a novel multi-dimensional index of wellbeing for households and across Africa and use Random Forest Machine Learning techniques to assess the importance score of households’ proximity to protected areas on their wellbeing outcomes compared with the importance scores of an array of other social, environmental, and local and national governance factors. This study makes important contributions to the conservation literature, first by expanding the ways in which wellbeing is measured and operationalized, and second, by providing additional empirical support for recent evidence that proximity to PAs is an influential factor affecting observed wellbeing outcomes, albeit likely through different pathways than the current literature suggests.

## Introduction

It is well-recognized that biodiversity loss and climate change are interconnected crises that require urgent action^1,2^. Protected areas (PA) are core tools for conserving landscapes that support biodiversity and provide climate-regulating ecosystem services^3^. The international community recently set an ambitious target of conserving 30% of the world’s ecosystems through some form of protected area designation^4^. However, as other scholars have previously demonstrated, PAs are increasingly expected to fulfill multiple other functions, including providing social benefits to surrounding communities^5^. A key aspect in this regard is that PAs support human wellbeing^6^. However, high conservation priority areas can also be areas with increased social conflict^7^. Conflict can undermine PA effectiveness on achieving environmental and social targets. This in turn can create feedback processes in which changes in ecological factors exacerbate social tensions and undermine wellbeing^8^. The resulting social conflict can have a reciprocal and negative impact on environmental and biological systems by destroying habitat, expediting landcover conversion, and even affecting evolutionary pathways for certain species^9^. Dynamics like this can impede PA effectiveness and jeopardize their ability to deliver social and environmental dividends^10,11^.

The effectiveness of PAs at conserving ecosystems and maintaining ecosystem services may hinge on their ability to deliver social dividends^12^. It is therefore critical to find ways to better understand the social and ecological dynamics surrounding PAs. In this study, we explore how proximity to PAs and related social and environmental factors impact observed wellbeing outcomes for surrounding households. Our study advances the conservation literature in two important ways. First, we operationalize and measure an expanded conceptualization of wellbeing that incorporates tangible and intangible components, which aligns wellbeing more effectively with the range of ecosystem services from which communities benefit. Second, we provide robust evidence for the importance of proximity to protected areas compared to other variables in affecting observed variance in household wellbeing outcomes using methods that are novel to the study of PA effectiveness. This study complements other related work that examines PA management and natural resource governance ^13^. As climate change and biodiversity loss continue to intensify and negatively impact human wellbeing, the scientific community must find ways to employ more effective conservation strategies, and this requires incorporating a wider range of indicators on social impacts into our measurement of conservation effectiveness^14^. This is increasingly urgent, as the impacts of the COVID-19 pandemic and subsequent economic shocks have highlighted the vulnerabilities and inequitable distribution of costs, risks, and exposure to natural and social hazards emanating from changes in ecosystem services^15^.

## Characterizing ‘wellbeing’ in the context of PAs

While the conservation community has become increasingly aware of the interplay between social and ecological factors as determinants of conservation success and failure, the metrics used to understand and quantify the social dividends of PAs have historically been limited to narrow measures such as household health outcomes, income, and education^16^. Recent assessments of the empirical literature suggest that most studies prioritize these socioeconomic and traditional development indicators as measures of social benefit^17^. Others have highlighted the glaring omission of subjective factors that are more closely attuned to cultural, spiritual, identity ecosystem services^14^. There is a pragmatic reason for that omission, as these have historically been the only widely available indicators included in large datasets like the Human Development Index or the Demographic and Health Survey (DHS). However, the omission of subjective wellbeing indicators limits our ability to understand the impact of PAs on a broader and more comprehensive conceptualization of wellbeing. For instance, a recent high-impact study measures the impact of household proximity to PAs across the global south using only DHS indicators of wellbeing^18^. Their justification of those indicators is based on a conceptual model that assumes the benefits communities receive from PAs are contingent on their ability to tap into revenue streams associated with tourism in the PA. While that may be true for some community members in protected landscapes, there are likely other pathways through which proximity to PA could influence wellbeing that are not accounted for in their conceptual model. Unfortunately, approaches like this impose a narrow set of assumptions across dramatically diverse communities, economies, political arrangements, and landscapes. As such, the models that are used to measure that impact have tended to impose linear and static associations among predictor and response variables that may be overestimated. While such studies have made important advancements in the field, they are limited by their assumptions and methodological constraints and subsequently may underestimate, overestimate, or inaccurately estimate the influence of PAs on social wellbeing.

While employing narrow indicators of social benefits can be useful for parameterization, operationalization, and hypothesis testing, it is important to consider social benefits more holistically. Prior studies have emphasized the need to understand social benefits through multidimensional measurement of wellbeing which includes objective and subjective dimensions and is impacted by environmental factors and governance^19^. The theory around wellbeing as a multidimensional phenomenon has vastly outstripped indicator development, but recent studies have developed subjective measures of wellbeing which include unique cultural values and attitudes, comparative measures including perceptions concerning other groups, aspects of equity and justice^20^^,^^21^ and self-actualization or satisfaction^22^. While there is still no standard definition of wellbeing, it is increasingly understood to incorporate several dimensions like those described in Table 1 that have been further elaborated in recent meta-analyses and review articles cited in the table.

**Table 1:** Dimensions of wellbeing.

Wellbeing dimension	Components	Description
Objective	Economic living standards; Health; Education	Objective wellbeing broadly refers to an individual’s basic needs and the factors that support those. These include indicators like income, health outcomes, educational attainment, physical security, etc. They are described well by McKinnon et al. (2016) and operationalized in empirical models by Fisher et al. (2021)
Subjective	Social Relations; Self-defined goals and pursuits; Perceived attainment of life quality and perceived threats like security	Subjective wellbeing refers to a wide array of factors that individuals use to define and pursue their wellbeing based on personal and cultural worldviews, lived experience, and other unique factors. Because they are amorphous and individually defined, McKinnon et al. (2016) demonstrate that they are underutilized in cross-national or multi-area studies. Fisher et al. (2021) demonstrate a practical approach to including subjective factors in empirical models
Comparative	Distributional factors; temporal changes; comparisons with reference groups and expectations	Comparative wellbeing refers to an individual’s expectations of their current wellbeing vis-a-vis reference points in history and future expectations as well as reference categories of other social actors or groups. Due to the perceptual nature of these expectations, comparative wellbeing may be thought of as a subcategory of subjective wellbeing. The aspects of comparative wellbeing are described elsewhere, for instance in Betley, et al. (2021); Corrigan, et al. (2018)
Equity/Justice	Inclusive governance; Empowerment; Freedoms; Procedural equity	Wellbeing is increasingly understood in terms of equity and justice for various social groups. This includes a range of governance factors and the inclusion of various groups in decision-making. These factors have objective, subjective, comparative, and legal definitions, and rich literature on environmental justice has been developed. Corrigan, et al. (2018) describe the salience of these factors for evaluating conservation effectiveness, and Fisher (2022) describes a framework to utilize procedural, distributive, and retributive justice as heuristics to evaluate the effectiveness of environmental governance
Ecological	Social-ecological relationships; Environmental health; Ecosystem integrity	The conservation literature is beginning to include ecological conditions and environmental integrity as components of wellbeing including anthropogenic disturbance, natural hazards, and rights of nature. The empirical literature that operationalizes such factors in wellbeing evaluation lags theory development. Ghoddousi et al. (2022) articulate the literature and conceptual connections, and Fisher et al. (2021) demonstrate ways to integrate such factors into empirical models

The use of diverse indicators that capture the multidimensionality of and the contextual nuance of wellbeing is confounded by the variety of spatial and social scales they operate in as well as the interconnectivity and dynamic feedback across dimensions. At the local level, this makes operationalization in empirical studies difficult due to inconsistencies regarding the directionality of influence and the lack of available data. At the regional or global level, designing models, harmonizing indicators and spatial scales, and doing so with variables for which data are accessible, has proven to be a daunting task. In this paper, we make methodological advancements by using a multidimensional indexing approach that enables comparison of indicators across diverse social, economic, and political contexts. Specifically, we utilize a robust indexing approach^32^ to create multidimensional indices of objective wellbeing, subjective wellbeing, and an overall composite of wellbeing at the household level using data from Afrobarometer household surveys^23^. The input parameters used to construct those wellbeing indices are included in Table 4 in the methods section below.

While traditional econometric and linear statistical methods have been previously used to assess the relationship between PAs and social outcomes, recent work suggests that machine learning approaches may be suitable complements and may be able to examine the influence and relative importance of large sets of predictor variables on observed human wellbeing outcomes more accurately^45^. Toward that objective, we utilize random forest regression machine learning models to examine the importance scores of household proximity to a PA and the size of the nearest PA as factors that affect movement in observed wellbeing outcomes compared to other non-PA related factors including landcover change, proximity to infrastructure, local and regional governance, exposure to stochastic shocks, and other predictors outlined in Table 5. While previous studies have utilized causal modeling approaches based on narrow conceptual models to demonstrate that proximity to PA is a predictor of better wellbeing, questions remain regarding how important proximity to PA is compared with other factors. The random forest regression modeling approach we employ fills that knowledge gap by utilizing a decision-tree based approach to determine the importance scores of predictor variables in affecting movement and distribution in observed wellbeing outcomes across observations. This complements other studies by demonstrating how important the proximity variable is relative to other factors in determining variance in observed wellbeing outcomes across households in our sample. This technique is limited, however, because the decision tree architecture does not directly estimate the size or direction of the correlations. Instead, it detects how consistently important the variable is in movement in the response variable. Based on the initial model outputs, we then test whether distance to PA and size of PA affect household wellbeing outcomes differently for households located within 10 km buffer of a PA compared to households outside that 10 km buffer following designs by^9^ and^18^. The construction of our response and predictor variables as well as the design of our empirical models is described more fully in the methodology section below.

## Results

**Figure 1:**
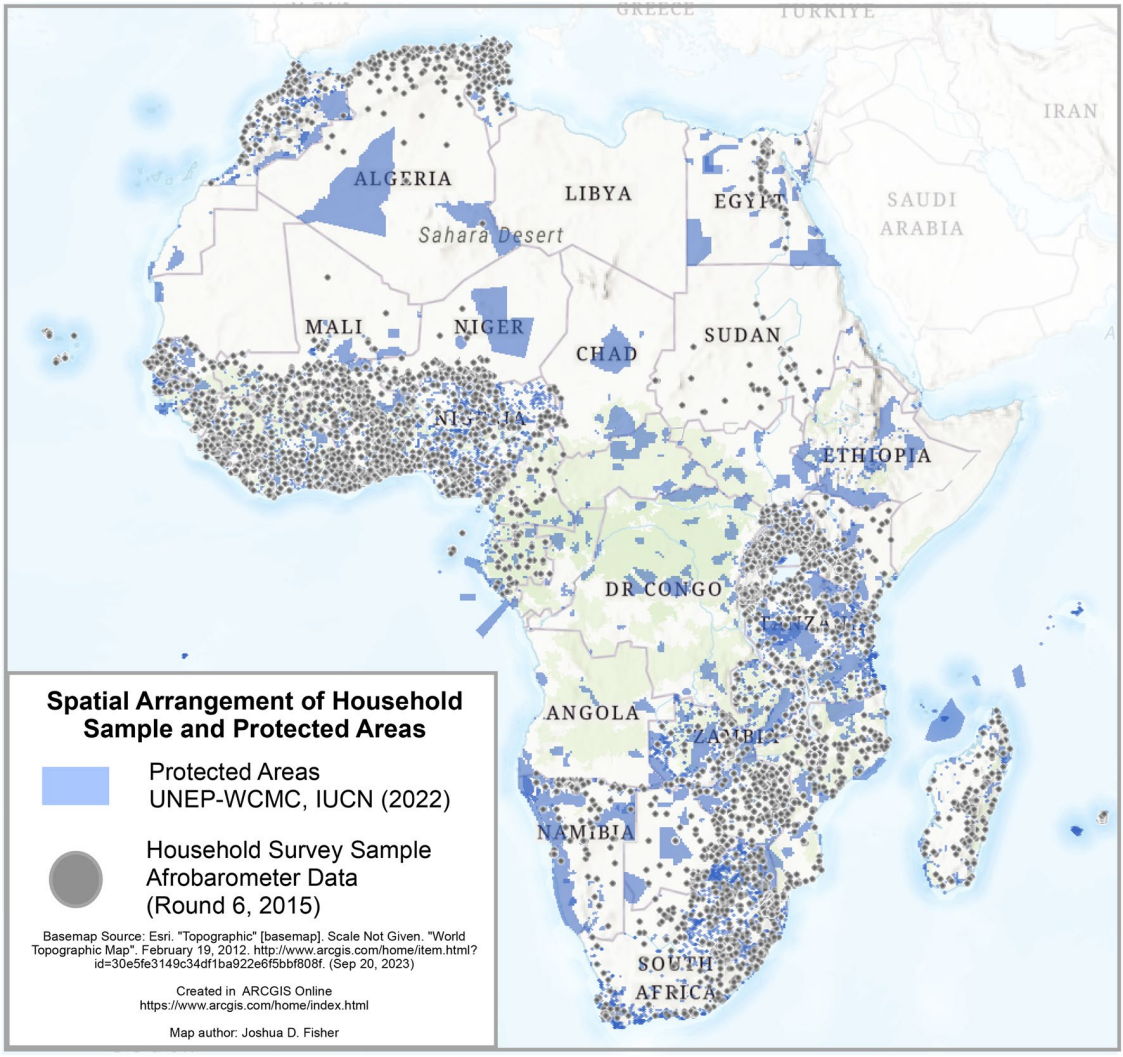
Distribution of Afrobarometer sample and protected areas.

Upon completion of our data cleaning, geoprocessing, and compilation of our composite wellbeing indices, we arrived at a total sample of *n* = *43,404* households to include in machine learning models of which 19,680 households were within 10 km of a PA. The geographic distribution of the data is presented in Fig. 1 below.

We estimate random forest regression models using three response variables: objective wellbeing composite, subjective wellbeing composite, and an overall wellbeing composite consisting of the indicators included in both objective and subjective composites. We include all predictor variables in each model to estimate the importance scores of each on movement in the response variables. Following the initial models, we divide our sample into two groups (within and outside a 10 km buffer of the nearest PA) to assess the relative importance of the predictor variables in each subset of the sample to further illuminate the role of proximity to PAs in affecting distribution of wellbeing outcomes. In assessing model fit, we include three measures: out-of-bag estimation (OOB) which calculates the prediction error of the random forest model based on the sample of decision trees, R^2^ which measures the amount of variance described by the model, root-square mean error (RMSE) which calculates the difference between the predictions for the outcome variable and the observations for the outcome variable. Each measure captures different information about the model, and collectively they provide a holistic picture of the model fit.

As shown in Table 2, the overall wellbeing model performed best (R^2^ = 0.391), with subjective wellbeing performing similarly (R^2^ = 0.378) and the objective wellbeing model not performing as well (R^2^ = 0.296). While these R^2^ values are lower than normally accepted values for linear models, the OOB and RMSE values are low in the models which indicates a high level of predictive accuracy. We implemented a variety of model specifications using subsets of variables and found that the OOB and RMSE were stable across multiple specifications while the R^2^ was more sensitive to the inclusion or omission of predictor variables. This indicates that while we are not describing the entire range of variables that affect wellbeing scores, the variables we do include are important in driving variation in distribution the observed wellbeing outcomes in our sample.

The importance scores of the predictor variables are presented in Table 2, with each model sorted on the importance scores from high to low for ease of interpretation. These importance scores indicate the relative importance of a variable compared to all other variables, but they do not indicate a positive or negative correlation with higher outcomes in the response variable. Comparing the models, there are noticeable differences in the importance scores and the position of the predictor variables in the sorted model outputs. Interestingly, however, each model has three relative groupings or tiers of importance scores. The top tier includes variables with importance scores that are much larger than the others, with importance scores consistently in above 100 in each model. The second tier in each model includes the majority of variables, with scores ranging broadly between 20 and 80 in each model. Finally, a lower tier includes variables that have relatively low importance scores for the overall model, typically below 20. While these tiers are not robustly defined, they are useful aggregations for discussing the differences between the various models.

The most useful comparisons among models are between the Objective Wellbeing and Subjective Wellbeing models, as these are intended to measure qualitatively different dimensions (see Table 2) of wellbeing. The Overall Wellbeing is a composite that includes all dimensions from both Objective and Subjective measures and is thus less useful for understanding the variables that play an important role in affecting observed outcomes for each type of wellbeing. The most notable distinctions between these models are found in the top and bottom tiers of the variable importance scores. For the Objective model, the most important variables are household facility access and educational attainment, both of which have been shown elsewhere to be positively correlated with objective wellbeing^18^. For the Subjective model, the most important variable is the respondent’s perspective on the direction in which the country is headed, which captures the general attitude the respondents have about the place in which they live and likely captures intangible aspects of their lived experience. In contrast, that same variable has low importance score for objective wellbeing, whereas the household facilities and educational attainment variables have only moderate importance for subjective wellbeing. This suggests that objective and subjective wellbeing are indeed discrete phenomena and have unique relationships and associations with the predictor variables.

**Table 2:** Afrobarometer Random Forest Models of Overall, Objective, & Subjective Wellbeing. Each model is sorted by importance scores from high to low.

Model	Overall wellbeing	Model	Objective wellbeing	Model	Subjective wellbeing
Perspective on the country’s direction	132.335	Household facility access (water, sewage, electricity)	116.093	Perspective on the country’s direction	496.424
Household facility access (water, sewage, electricity)	78.146	Educational attainment	111.497	Standard deviation of NPP	84.649
Educational attainment	69.39	Change in lights coverage	67.537	Change in crop coverage	84.246
Change in tree coverage	42.901	Change in tree coverage	54.857	Change in lights coverage	84.05
Standard deviation of NDVI	41.44	**Distance to PA**	**52.159**	Household facility access (water, sewage, electricity)	83.917
Change in crop coverage	39.767	Change in crop coverage	51.816	Change in tree coverage	83.042
Standard deviation of NPP	39.704	Standard deviation of NDVI	51.516	Standard deviation of NDVI	83.006
Distance to roads	38.715	Standard deviation of NPP	51.318	**Distance to PA**	**82.292**
**Distance to PA**	**38.564**	Distance to roads	51.169	Representative government	81.51
**Size of PA**	**37.545**	Village/municipal facilities availability (water, sewage, utilities, cell coverage)	50.314	Distance to roads	78.308
Change in urbanization	37.52	**Size of PA**	**50.225**	Change in urbanization	77.791
Change in lights coverage	33.004	Change in urbanization	49.441	Educational attainment	77.791
Distance to buildings	32.196	Distance to buildings	46.792	**Size of PA**	**77.582**
Village/municipal facilities availability (water, sewage, utilities, cell coverage)	30.936	Representative government	39.182	Distance to buildings	55.91
Representative government	29.758	Perceived Security	38.808	Floods	52.385
Perceived Security	29.06	Freedom of Speech	34.179	Perceived Security	48.093
Freedom of Speech	23.499	Income group of country	30.115	Freedom of Speech	46.22
Floods	22.362	Floods	29.449	Voting freedom	34.43
Income group of country	19.031	Voting freedom	25.921	Village/municipal facilities availability (water, sewage, utilities, cell coverage)	34.272
Voting freedom	16.987	Threats	23.437	Threats	28.481
Threats	15.727	Perspective on the country’s direction	18.442	Droughts	26.028
Droughts	13.483	Droughts	16.69	Income group of country	25.058
Physical Security	10.289	Physical Security	16.373	Physical Security	19.99
Extreme Temperature	2.847	Extreme Temperature	3.452	Extreme Temperature	5.32
***N***	***43,404***	***N***	***43,404***	***N***	***43,404***
***OOB Error***	***0.138***	***OOB Error***	***0.02***	***OOB Error***	***0.03***
***R***^***2***^	***0.391***	***R***^***2***^	***0.296***	***R***^***2***^	***0.378***
***RMSE of prediction model***	***0.118***	***RMSE of prediction model***	***0.177***	***RMSE of prediction model***	***0.172***

The models each locate size of and distance to PA as having moderate importance as predictors of household wellbeing scores. The key variables of interest (Distance to nearest PA and Size of nearest PA) are presented in bold text for ease of comparison across models. Model fit statistics (*Out of Bag Error*, *R*^*2*^, and *Root Mean Square Error*) are provided in bold and italicized text.

After accounting for those variables with high or moderately high importance scores, the next set of variables for both the objective and subjective models are interesting, as many of the variables with moderate importance scores involve changes in land cover (crops, trees, urbanization, measures of productivity such as NDVI and NPP), and importantly, the absolute distance of a household to the nearest PA as well as the size of the nearest PA. There are interesting nuances in each model, with village-level facility availability playing a more important role in determining variance in objective wellbeing outcomes, and government representativeness of household concerns playing a more important role in affecting movement across subjective outcomes. However, again the common theme among the second tier of variables for both models is that landcover change, environmental, and geographical variables have moderate importance scores in terms of their effect on movement in observed wellbeing outcomes. There is a steep decline in importance scores for the remaining variables which include anthropogenic environmental threats, stochastic climate shocks, granular local and national political or governance factors, and the overall macroeconomic context. This indicates that these variables play only a limited role in the outcomes as modeled in the underlying decision trees.

**Figure 2:**
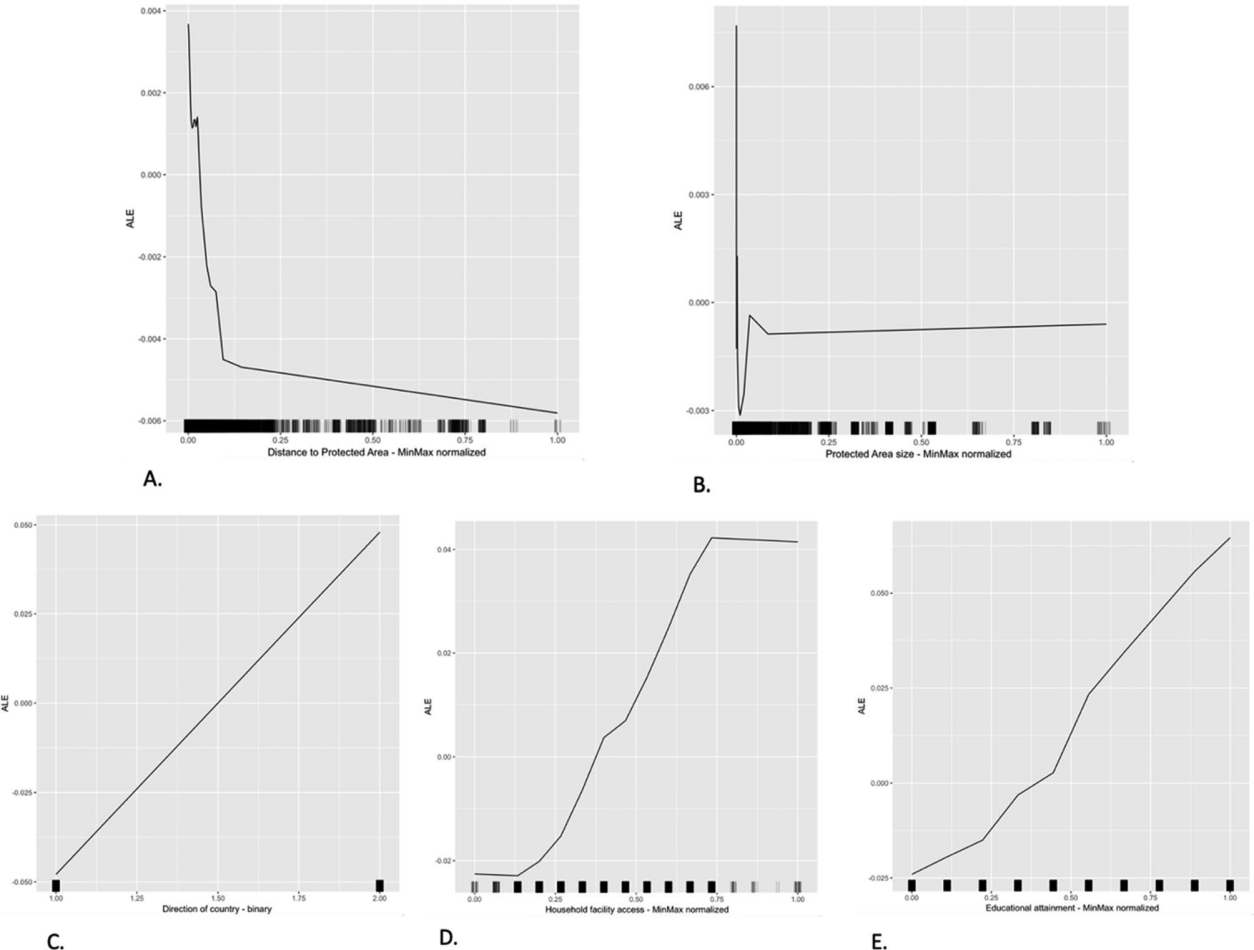
ALE plots for: (**A**) Distance to PA, (**B**) Size of PA, (**C**) Direction of the country, (**D**) Household facilities access, and (**E**) Education attainment of the respondent. (**A** & **B**) demonstrate that distance to PA and size of PA both operate in the anticipated directions. Larger distances (horizontal axis) are associated with lower importance scores for overall wellbeing (vertical axis). Likewise, larger PAs are positively associated with higher importance scores. (**C**, **D**, & **E**) demonstrating that the association between the most important predictors in the overall wellbeing model and the direction of the relationship behaves as expected. (**C**) demonstrates that higher wellbeing scores are associated with reports of the country moving in the ‘right direction’. (**D**) shows that higher levels of household facilities (water, electricity, sanitary facilities) are associated with higher wellbeing. Outcomes (**E**) demonstrates that higher education levels correspond to higher wellbeing outcomes.

As discussed earlier, we are primarily interested in understanding the role that proximity to PA and PA size play as influencing movement or variance in observed household wellbeing outcomes. The model outputs described above demonstrate that those two variables have moderate importance scores across our three measures of observed wellbeing. Previous studies that use causal modeling techniques have shown a positive relationship between proximity to PA and higher wellbeing outcomes, While our modeling approach does not allow us to explicitly test that directional relationship, we were interested to see how importance scores varied across the range of observed values in the proximity to PA and size of PA variables. This would enable us to examine whether the importance scores are higher for households nearer to PAs and for households near larger PAs, assuming that such households may have more ready or more reliable access to the ecosystem services of those protected landscapes. To analyze this, we constructed accumulated local effects (ALE) plots for those two variables, as well as for the other predictor variables with the highest importance scores in our overall wellbeing model (Fig. 2). Those plots facilitate ease of interpretation for machine learning models by centering importance scores between -1 and 1, then plotting the importance across the variable’s measured range.

As anticipated, both size of PA and proximity of households to PA operate as we assumed. Regarding the distance measure, there is a steep decline in importance as distance from the PA grows, though the rate of decline tapers in the larger values of the distance variable. This may be due to a variety of underlying mechanisms that are explored further in the discussion section. The size variable shows an interesting pattern, where size has an immediate steep decline, followed by a steep fluctuations, then leveling off to an upward trend toward the larger values in the variable. This may be due to the large size differences in PAs across the sample, ranging from small urban PAs to expansive wilderness areas. Generally, however, the trend is for larger PAs to be associated with larger importance scores as drivers of wellbeing outcomes. In contrast, the ALE plots for the highest importance predictors of overall wellbeing are clearer, with sharp increases in importance scores associated with higher values of educational attainment, household facilities access, and the general direction the country in which the country is headed. For reference, we include ALE plots for all variables in the Supplemental Materials file attached to this study.

As the ALE plot for distance to PA (Fig. 2, *box A*) shows, there is sudden change in the trajectory of the association between distance and importance score as distance increases. To better understand the relationship of distance to PA and movement in observed wellbeing outcomes we examined the absolute distance variable in subsets of the sample stratified according to whether the household was within a 10 km buffer of the protected area or outside the 10 km buffer and estimated random forest models on both samples (Table 3).

**Table 3:** Random forest model outputs for subsets (within 10 km of PA and outside 10 km of PA) of the Overall Wellbeing index.

Model	≤ 10 km from PA	Model	> 10 km from PA
Perspective on country’s direction	53.88	Perspective on country’s direction	75.902
Educational attainment	33.098	Household facility access (water, sewage, electricity)	46.103
Household facility access (water, sewage, electricity)	32.501	Educational attainment	37.008
Change in lights coverage	18.85	Change in tree coverage	24.891
Change in crop coverage	18.276	Change in lights coverage	23.538
Change in tree coverage	17.691	Standard deviation of NDVI	22.097
Standard deviation of NDVI	17.691	Standard deviation of NPP	21.419
Standard deviation of NPP	17.251	**Distance to nearest PA**	**21.03**
Distance to roads	16.931	Distance to roads	20.658
Change in urbanization	16.862	Change in crop coverage	20.478
**Size of nearest PA**	**16.662**	Village/municipal facilities availability (water, sewage, utilities, cell coverage)	20.382
**Distance to nearest PA**	**15.293**	Change in urbanization	19.348
Distance to buildings	13.778	**Size of nearest PA**	**19.335**
Perceived Security	13.525	Representative government	18.122
Representative government	13.361	Distance to buildings	17.192
Village/municipal facilities availability (water, sewage, utilities, cell coverage)	11.825	Perceived Security	16.735
Freedom of Speech	10.472	Freedom of Speech	14.261
Flooding	9.674	Flooding	12.407
Income group of country	8.827	Income group of country	10.999
Voting freedom	7.842	Voting freedom	10.069
Threats	7.599	Droughts	8.238
Droughts	5.24	Threats	7.841
Physical Security	4.719	Physical Security	5.998
Temperature extreme	1.335	Temperature extreme	1.344
***N***	***19,680***	***N***	***23,724***
***OOB Error***	***0.014***	***OOB Error***	***0.014***
***R***^***2***^	***0.358***	***R***^***2***^	***0.413***
***RMSE of prediction model***	***0.014***	***RMSE of prediction model***	***0.12***

Table sorted on importance factors from high to low. The key variables of interest (Distance to nearest PA and Size of nearest PA) are presented in bold text for ease of comparison across models. Model fit statistics (*Out of Bag Error*, *R*^*2*^, and *Root Mean Square Error*) are provided in bold and italicized text.

**Figure 3:**
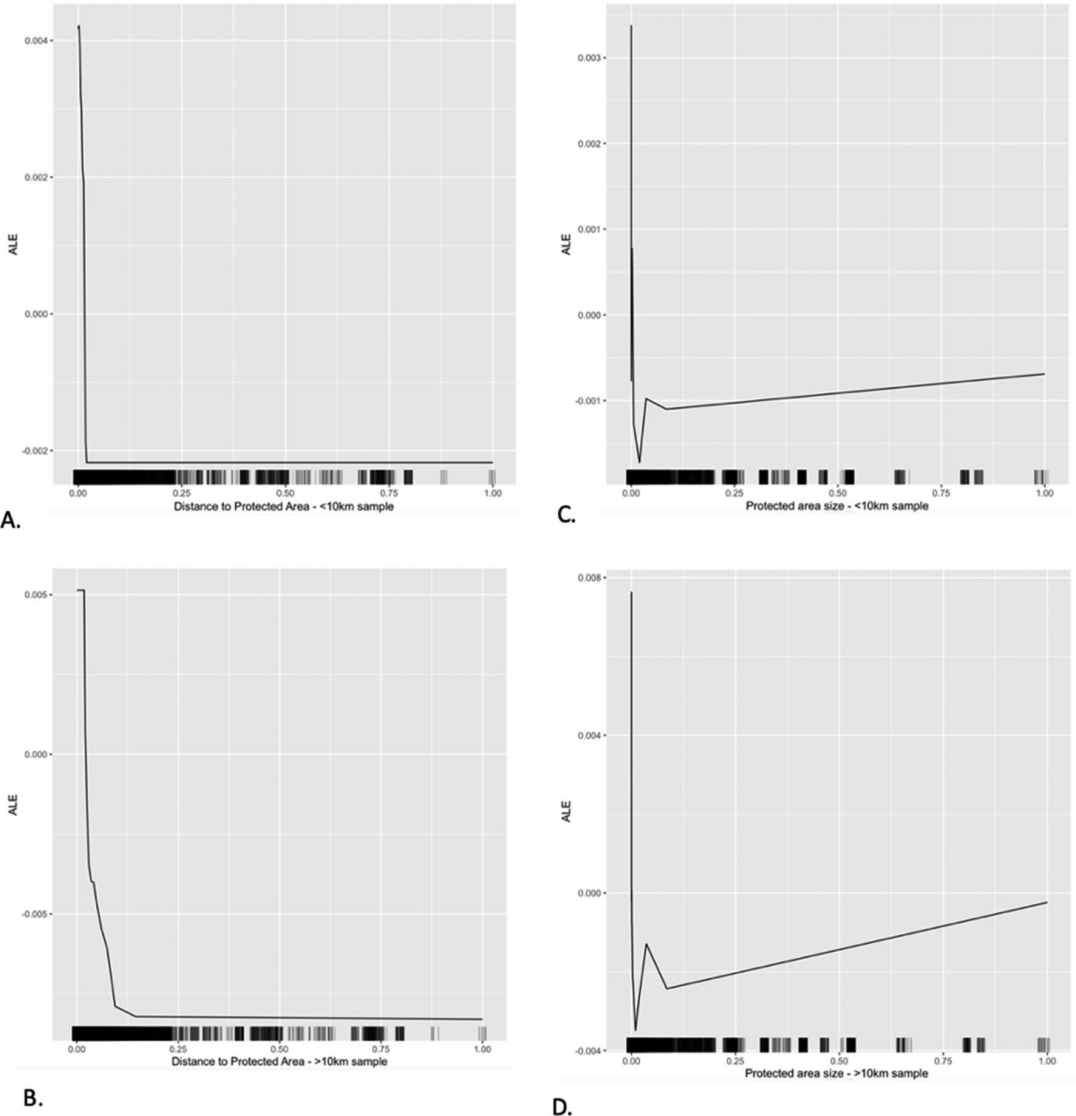
ALE Plots of subsets of the sample (within 10 km of PA and outside the 10 km buffer of PA) of the Overall Wellbeing index. ALE plots include (**A**) Distance to PA within the 10 km buffer, (**B**) Distance to PA outside the 10 km buffer, (**C**) Size of PA within the 10 km buffer, and (**D**) Size of PA outside the 10 km buffer.

In both models, the impact of PA size and household distance to PA maintain moderate importance scores relative to other variables. Interestingly however, the importance score of proximity to the PA does differ within the buffer zone and outside of it (Fig. 3). Within the buffer zone, distance from the household to the PA has a lower overall importance score relative to other variables and appears lower in the sorted table than it does outside the buffer zone. This indicates that closer to the protected area (within the 10 km buffer), absolute distance to the protected area has less importance on movement in observed wellbeing scores than it does outside the buffer zone. Interestingly again, within the buffer zone the size of the PA has a higher importance score than distance, whereas outside the buffer zone distance has a higher importance score than size. However, in the models for both subsets of households the same pattern is repeated as in the first model where socioeconomic and environmental and geographical factors are the highly important and moderately important factors affecting movement of observed wellbeing outcomes compared to stochastic shocks and local and national governance factors.

## Discussion

The approach we take in this paper is to use novel and more comprehensive measures of wellbeing than are typically employed in the conservation literature to examine the importance of proximity to PAs as a variable that drives movement of observed household wellbeing outcomes. Our approach to measuring wellbeing fills important gaps identified by^14^ and^17^, and our modeling contributes important empirical evidence to the work of previous studies that examine the influence of PAs on surrounding communities, such as^18^. Both contributions open many avenues for further investigation and exploration including underlying causality, the mechanisms through which PAs deliver social benefits, multidirectional influence of variables, and more. These are future strands of research that we aim to explore in follow-on studies. However, for the purposes of the current study we are principally focused on advancing the operationalization of multidimensional wellbeing in conservation studies and employing new methods to investigate the effect of proximity to PAs on social outcomes.

The results from our models complement previous findings that proximity to PA^18^ and size of PA^44^ are moderately important factors that impact movement in wellbeing outcomes at the household level compared to an array of other variables that have been explored in other studies including landcover change, governance, and socioeconomic factors. However, distance to and size of the nearest PA are not as important in determining movement in the wellbeing response variables as other studies have suggested^18^, and socioeconomic factors including the household economic situation, educational attainment, and the general direction of the country are by far the most important factors that influence movement of wellbeing outcomes in our machine learning models. The influence of socioeconomic factors is sufficiently high in the random forest models to support our earlier speculation that linear models used in other studies may be overestimated due to the importance of socioeconomic factors. Our model fit R^2^ parameter was sensitive to the inclusion and omission of these factors, while the importance scores of our predictor variables, and OOB and RMSE model fit parameters were relatively stable over various specifications. The fit of linear models is likely highly dependent on these variables, and omission might significantly alter the fit and overall significance and confidence of covariates. With this in mind, our models demonstrate the moderate importance of the distance to PA variable and size of PA variable in our random forest regression model. This provides important evidence of the influence of those variables in driving movement of observed scores in the response variable. This in turns provides important empirical evidence that those variables should be included in models that seek to further illuminate causal relationships among PAs and wellbeing. While we did not explicitly examine that causality or the underlying directionality, we believe this is an area that future research in the field could explore more thoroughly.

Additionally, local environmental factors like land cover change are also important predictors of movement in observed household wellbeing outcomes, many of which have higher or roughly equivalent importance scores than the proximity and size measures in our models. Interestingly, the stochastic climate shocks and anthropogenic threats included in our models appear to have a much lower influence on movement in observed wellbeing outcomes. When these results are taken together, an interesting picture begins to emerge. Considering the high prevalence of agricultural and natural resource dependent economies in the geographies represented in the Afrobarometer sample, it is logical to assume that the livelihoods associated with various value chains in those geographies are impacted by changes in local environmental conditions. Additionally, the maintenance of ecosystem function and ecosystem service production likewise should logically impact livelihoods in these geographies. In that sense, it follows that PAs should be major factors influencing wellbeing outcomes precisely because their purpose is to safeguard those systems and build resilience to exogenous shocks. However, the exact relationship between a given PA and a given household near that PA is likely influenced by myriad factors, including household income streams and composition of livelihood portfolios, the type of interactions and interdependencies that household has with the ecosystem and biodiversity of the PA, and others. For agrarian households, the proximity to certain species may heighten human wildlife conflict and thereby reduce wellbeing. Other households may depend on the ecosystem for temperature and hydrological regulation and thus have elevated wellbeing scores. Still other households may have idiosyncratic relationships where objective wellbeing is high and subjective wellbeing is low, or vice versa. Because of such idiosyncrasies, our study makes an important contribution in demonstrating that proximity is in fact important in the overall set of factors that affect wellbeing outcomes without imposing static or uniform assumptions related to the nature and directionality of that influence.

Interestingly, certain subjective factors are more important for influencing wellbeing outcomes than more objective factors. For instance, conflict and security are important factors affecting wellbeing outcomes^24^. We included an explanatory variable of the perceived security situation for households as well as a more objective measure of whether the household or its members had experienced an incidence of violence or conflict during the past year. Across all models, the perceived security situation was a more important indicator than the recent experience of conflict. This reinforces the notion that uniform assumptions around the influence of objective measures should not be imposed across geographies, ecologies, or cultures. Rather, in assessing household wellbeing, we need a more nuanced and tailored approach to unpack the connections and influence of predictor variables.

It is important to note that the Afrobarometer survey is designed and conducted to assess governance across countries in Africa. The sample of countries included in the Afrobarometer is not comprehensive, and there are likely political, economic, and security reasons for that. This potentially introduced a spatial bias into our modeling results that is unavoidable. However, other studies^18^ employ a much broader geographic sample using DHS data on to examine narrow definitions of wellbeing. Our results complement those previous results, suggesting that the potential spatial bias may not be a great concern. Where previous studies have tied the social benefit of PAs directly to the ability of a household to access income derived from the PA through tourism or other revenue flows, our results hint toward the interconnectivity of socioeconomics, landcover change, and governance, with PAs and intact landscapes being vehicles through which ecosystem services are maintained and benefits captured. However, our results are limited in that they are not able to elucidate the specific mechanism or explicit connections between these factors in generalizable ways. That requires a different methodological approach that can work within specific cultural, environmental, and political contexts.

Another important limitation of the current study is the lack of disaggregated models that examine the influence of locally defined contextual factors on wellbeing outcomes. As^43^ and others have articulated, the relationship between communities and PAs is highly contextually defined and nuanced. The scientific community cannot assume that the mechanisms through which communities derive value and wellbeing from PAs is the same across geography, political and socio-economic contexts, or management approaches. For example, there are likely different underlying relationships, mechanisms, and from a modeling perspective there may be different directions of influence in the statistical relationships among wellbeing and PA proximity for communities neighboring a national park in Latin America as those neighboring a private or community managed PA in Africa, in the sub-Saharan region or in distinct locations in either context. In this study, we focus primarily on advancing the methodological approach to measuring wellbeing and building an empirical evidence base for the relationship between PAs and movement in observed wellbeing outcomes. In so doing, we omit disaggregated models to avoid paying insufficient attention to the local context. However, we have begun to explore those relationships in complementary studies^13^ and aim to do so in future studies as well. Importantly however, there is a growing literature examining whether IUCN categories and other typologies of PA categorization are useful or empirically robust methods of assessing PA effectiveness^44^. Such questions are outside the scope of the current study but warrant further dedicated studies. However, such studies suggest that size of PA may play an important role in PA effectiveness, and again one contribution of this study is that it provides empirical justification for the inclusion of such variables in future studies.

While previous work has demonstrated a net-positive impact of proximity to PAs for households across portions of the global south^18^, those studies have employed narrow definitions of wellbeing that were limited to traditional socio-economic factors that capture only a narrow slice of the contemporary understanding of wellbeing’s multidimensionality. Such studies employed at the global level also impose a rigid set of assumptions between covariate and response variables that might not hold up to scrutiny when examining a given geography with nuanced cultural, political, and environmental contexts. Despite the limitations mentioned above, our results indicate that PAs are important factors in influencing multidimensional wellbeing outcomes. As calls for expanding PAs globally are being translated into policy^4^, it is important that the scientific and conservation communities develop a better understanding of how to manage PAs to deliver both social and ecological benefits, avoid stakeholder conflicts, and maximize the potential of these areas to deliver the types of outcomes that can build resilience against our world's impending challenges.

In this paper, we expand the definition and operationalization of wellbeing to include both objective and subjective components. Rather than using individual metrics as proxies for wellbeing writ large, we develop statistically robust composite indices that capture a more holistic view of wellbeing. The inclusion of subjective measures, self-reported by households across a wide array of cultural, political, and geographic contexts, enables us to fill an important gap identified by previous studies while also enabling us to circumvent the imposition of static assumptions across culturally nuanced and distinct households.

Importantly, our study highlights two important directions for future research. On one hand, the global community must continue to expand the definition of wellbeing that is employed in both practice and policy around PAs. Across and within communities, there exist important differences in the ways community members interact in and with the natural environment. Designing an effective PA management strategy depends on working collaboratively with various groups of stakeholders to understand their unique and self-defined interests, needs, and goals. Additionally, our frameworks for evaluating PA effectiveness must move beyond the imposition of uniform and static assumptions to be more dynamic, more adaptive, and more agile for changing conditions and interconnectivity across social-ecological systems. Over recent decades, previous work by other scholars, practitioners, and communities has made great strides in advancing the methods, tools, and practices of conservation and effective natural resource management. Continued support and collaboration across those groups will be ever more crucial to effectively confront the pressing social and environmental challenges our world faces.

## Methods

As noted earlier, previous studies have demonstrated that proximity to PAs has a net positive impact on wellbeing defined in socioeconomic terms based on narrow conceptual models and the imposition of strict assumptions that are likely not applicable across diverse cultural and ecological contexts. Recent studies have emphasized the need to understand social benefits and wellbeing more holistically to include some measure of objective wellbeing (socioeconomic indicators), subjective wellbeing (self-defined and comparative indicators), environmental wellbeing (ecological indicators), and governance (governing, management, and equity and justice indicators)^19^. Other research demonstrates how these various components can be operationalized empirically at the country level, but to date, we are not aware of any studies that systematically incorporate these components into empirical studies of PA effectiveness in systematic and scalable ways, particularly at sub-national or spatially explicit levels^24^.

We begin to address that gap by constructing a multidimensional, composite index that includes both objective and subjective measures of household wellbeing as a response variable. We then construct empirical models to examine the impact of predictor variables on the composite index, including local and national governance factors, environmental changes and shocks, and a variety of physical or geographic factors. Importantly, because we understand that many social-ecological relationships are highly context-dependent, we do not impose strict linear assumptions on the relationships between predictor and response variables. We employ random forest regression machine learning techniques^25^ to assess the importance of variables in influencing movement and distribution of observed wellbeing outcomes across our sample. The use of these methods is gaining traction in the conservation community given their flexibility for potentially correlated variables and nonlinear relationships. Recent studies have employed random forest regression and classification to problems such as deforestation^42^, soil quality^26^, tourism and recreation^27^, and erosion prediction^28^. To our knowledge, these techniques have not yet been applied to protected area effectiveness studies nor the social impacts of protected areas. As such, the use of random forest regression in this study is novel compared to the classical linear models and more recent Bayesian models that have become standard in the conservation literature.

### Constructing the response variable

To construct an expanded indicator for wellbeing, we build off the foundation of an earlier study^24^ to construct a composite index of objective and subjective wellbeing. Previous studies in the conservation literature use Demographic and Health Survey (DHS) data to measure objective wellbeing using individual indicators of economic, health, and education as proxies for wellbeing^18^. Each of those could be considered a key component of objective wellbeing, but when considered in concert they present a more well-rounded or comprehensive picture of objective wellbeing. As discussed earlier, subjective wellbeing refers to individually defined indicators of wellbeing and comparative assessments of actual performance against expected outcomes. These are typically culturally or locally nuanced, making it difficult to construct indicators that are effective across contexts. Previous work describes these nuanced facets of wellbeing^14^^,^^17^. In previous research, we circumvented that difficulty by constructing indices of individuals’ self-assessment of their life satisfaction which asks respondents to rank their overall satisfaction with their life and living situation, as well as their life evaluation which asks participants to compare their life now to the past, the future, and others^24^. Such historical, present, and comparative self-assessments have elsewhere been shown to be important factors affecting wellbeing, albeit in the context of sustainably peaceful societies^46^. Using a similar rationale, we understand subjective wellbeing to be nuanced and multidimensional and to include aspects of comparative wellbeing regarding historical, future, and distributional components. We also understand that, like objective wellbeing, no single indicator is an effective proxy for this construct, but rather a mosaic of related constructs provides a richer and more comprehensive view of subjective wellbeing. We, therefore, opted to construct composite indices of objective and subjective wellbeing using variables like those in previous studies, and then combine them into an overall wellbeing composite. The input parameters for our composite indices of objective, subjective, and overall wellbeing are described in Table 4.

**Table 4:** Composition of composite wellbeing indices.

Composite index	Components	Afrobarometer question #	Afrobarometer question	Scale
Objective wellbeing	Present living conditions	q4b	In general, how would you describe your own present living conditions	5-point Likert
	Food security	q8a	Over the past year, how often, if ever, have you or anyone in your family gone without enough food to eat?	4-point rank
	Asset based wealth	q91	Which of these things do you personally own? (Radio, Television, Motor vehicle/motorcycle, mobile phone	Sum
	Health security	q8c	Over the past year, how often, if ever, have you or anyone in your family gone without medicines or medical treatment?	4-point rank
Subjective wellbeing	Comparative living conditions	q5	In general, how do you rate your living conditions compared to those of other[s]	5-point Likert
	Past perception of country economy	q6	Looking back, how do you rate economic conditions in this country compared to twelve months ago?	5-point Likert
	Current perception of country economy	q4a	In general, how would you describe the present economic condition of this country?	5-point Likert
Overall wellbeing	Present living conditions	q4b	In general, how would you describe your own present living conditions	5-point Likert
	Food security	q8a	Over the past year, how often, if ever, have you or anyone in your family gone without enough food to eat?	4-point rank
	Asset based wealth	q91	Which of these things do you personally own? (Radio, Television, Motor vehicle/motorcycle, mobile phone	Sum
	Health security	q8c	Over the past year, how often, if ever, have you or anyone in your family gone without medicines or medical treatment?	4-point rank
	Comparative living conditions	q5	In general, how do you rate your living conditions compared to those of other[s]	5-point Likert
	Historic recall of country economy	q6	Looking back, how do you rate economic conditions in this country compared to twelve months ago?	5-point Likert
	Current perception of country economy	q4a	In general, how would you describe the present economic condition of this country?	5-point Likert

Unfortunately, few surveys or existing datasets include questions relevant to both objective and subjective indicators that are also readily converted into the spatially explicit formats needed to explore the impact of proximity to PAs. This prevented us from using the same data that other studies have employed. However, the Afrobarometer survey is an exception in that it includes questions relevant to both objective and subjective wellbeing and is administered using similar protocols to each other across a wide range of countries in their respective geographic regions on similar periods. It is a public opinion survey that is conducted annually and covers topics ranging from personal security, education, infrastructure, and living conditions. It collects information on a variety of factors relevant to objective and subjective wellbeing albeit using different enumeration protocols and variations in questions and responses. The Afrobarometer data are available as cleaned and geocoded data^29^. In this study, we utilize data from Round 6, which was implemented in 36 countries in 2014–2015. These data were provided under academic license for this study.

In selecting variables to include in the objective and subjective wellbeing composites, we could not assume that variables of interest were missing at random and therefore, the analysis was restricted to indicators with less than 5% missing values. To capture objective wellbeing, we utilized the following: the respondent’s employment status, food security in the household; access to health care; and a composite measure of asset-based wealth. To capture subjective wellbeing measures, we utilized the following: household wellbeing in comparison to other community members; household wellbeing compared to previous wellbeing; and how households viewed their current living situation. Given the likely multidimensionality in any measure of wellbeing, a composite index is useful to capture a range of indicators^30^. Our variables of interest were not correlated and could not be assumed to be substitutes. Previous literature has documented the potential constraints of assuming the components of poverty indices are compensatory^31^. Therefore, rather than factor analysis, we ensured our composite measures were reliable using Chronbach’s alpha measurement and created indices for objective wellbeing and subjective wellbeing utilizing a generalized non-compensatory method, the Mazziotta-Pareto Index^32^.

### Compiling predictor variables

As noted above, much of the contemporary work assessing the effectiveness of PAs has employed traditional development indicators that have been shown to highly correlate wellbeing outcomes. These have typically included elements of income, health, and education. Other studies and frameworks such as the Protected Area Management Effectiveness toolkit^33^ focus on exploring the relationship between local or national-level governance and PA outcomes, while others have explored the impact of geophysical changes and climate-induced shocks. The evidence base and rationale for the inclusion of a variety of predictor variables have been thoroughly discussed in these and other studies and are by now commonplace in the conservation literature^18^^,^^20^^,^^21^. However, the empirical studies that operationalize those variables work on somewhat different and typically overly simple conceptual models that are based on narrow sets of generalized assumptions around causality, influence, and importance of some variables over others. For instance, the model that underpins^18^ is based entirely on a model that assumes PA benefit to surrounding communities depends on tourism revenue from the PA, and thereby omits other mechanisms through which societies might benefit. However, other mechanisms could logically include the safe and reliable production or delivery of a range of ecosystem services that are not necessarily monetary in nature or monetizable. The reliance on such simplistic causal models presents challenges for designing a conceptual model that works across contexts and selecting a suite of indicators that work across geographic, political, and cultural boundaries. Moreover, the pragmatic constraints of data that measure such indicators present real barriers to the development of precise and refined indicators. We, therefore, balance conceptual clarity, empirical justification, and pragmatism in selecting variables.

For the present study, we are primarily interested in understanding the importance of proximity to PAs and size of the nearest PA relative to other variables, and as such include a measure of distance to the nearest IUCN PA as well as the geographic size of the nearest PA using data from the World Database on PAs^34^. In addition, we understand PAs as social-ecological systems that are nested in wider social, political, economic, and environmental systems, each with its own sets of dynamic feedback processes tied to local conditions and context^35^. We know from previous empirical studies cited above that various categories of indicators influence wellbeing outcomes (socio-economic, political/governance, environmental geographic, and stochastic shocks) for households in the study geographies. We therefore include measures for each category to assess their influence on wellbeing outcomes. The data used to measure predictor variables is described in Table 5. To assign values of each variable to a specific household observation, we employed the following order of operations. Socio-economic data and governance indicators are sourced primarily from the Afrobarometer data, and thus already associated with a particular household. We recoded each variable such that answers like ‘not reported’ and ‘unanswered’ were changed to ‘missing’. As with construction of the response variable, we only included predictor variables in our study that had no more than 5% of ‘missing’ observations. We assume that the relationship between wellbeing outcomes and environmental factors is nuanced and complex, and that environmental and social factors operate on different timescales. We also assume from a large body of theoretical literature, much of which is described in^8^, that social impacts of protected areas and other conservation strategies result from changes in the natural world. As such, we observed environmental factors over extended time periods that include the 2015 reference year but extend before and/or after. For the study, variables expected to influence movement in wellbeing outcomes included control variables for the income group of the country, the occurrence environmental shocks including drought, floods, and extreme temperature, the micro-economic situation of the household (either according to the respondent or observed by the enumerator), the presence of village facilities (water, electricity, sewage, and cell service), the distance to the nearest PA, the size of the nearest PA, and relevant indicators at the household level including the respondent’s perspective on the direction of the country, household facility access (water, sewage, electricity), the household head’s educational attainment, perceived security, physical security, freedom of speech, voting freedom, and representative government. While management and governance of the PA are expected to influence social outcomes^13^, we were not able to include information on the governance of the PA due to lack of granular data in the World Database of Protected Areas^52^ and large numbers of missing values for IUCN PA type in the dataset.

We assume that some aspects of landcover and landcover change should influence movement in observed wellbeing outcomes, so we constructed metrics that measure change over time. Due to data limitations preceding the survey year, we utilized a period of 2015–2019, assuming that land cover changes in this period are representative of longer-term trends (aside from stochastic shocks). We controlled for factors expected to influence the heterogeneity in these groups including the standard deviation of net primary productivity and NDVI from 2015 to 2019, and the change in the following spatial variables between 2015 and 2019: crop cover, urbanization, nighttime illumination, and tree cover. We also assume that connectivity is important and include distances to the nearest road and buildings. Additionally, following^37^, we assume exposure to anthropogenic ecological threats could be an important factor affecting wellbeing and include a measure of this. Environmental measures were sourced from a variety of remote sensing data and preprocessed geospatial data described in Table 5 and processed using Google Earth Engine^36^. For variables that involved averaging or taking standard deviation over time, the period was 2015–2019. To assign values of each variable to a household observation, we assigned the value of the pixel at which the household is located, given its spatial coordinates. In the event that multiple pixels overlapped a household’s coordinates, we averaged across those values. Given the various ranges of the ordinal variables included, we utilized min–max techniques to normalize the variables included in our models. Two exceptions to that normalization are noted in Table 5.

**Table 5:** Data sources and transformations for predictor variables.

Predictor	Description	Transformations	Source	References #
Income group of country	Income categories of World Bank in 2015	Quartile scaled (low income, medium–low income, medium–high income, high income)	World Bank, 2015	^56^
Perspective on country’s direction	Perceptions of whether country heading in wrong or right direction	Binary	Afrobarometer, q3	^23^
Village facilities (water, sewage, utilities, cell coverage)	Type of infrastructure available in village	MinMax Normalized	Afrobarometer, EA_SVC_A to EA_SVC_D	^23^
Household facility access (water, sewage, electricity)	Type of infrastructure accessible to household	MinMax Normalized	Afrobarometer, q93, 93b, 94	^23^
Change in lights coverage	2015–2019 average change in nighttime lights at pixel level where household is located	MinMax Normalized	Image and data processing by Earth Observation Group, Payne Institute for Public Policy, Colorado School of Mines DMSP data collected by US Air Force Weather Agency	^48^
Change in tree coverage	2015–2019 average change in tree coverage at pixel level where household is located	MinMax Normalized	Hansen, et al., 2013	^49^
Change in crop coverage	2015–2019 average change in crop coverage at pixel level where household is located	MinMax Normalized	Copernicus Sentinel data, 2015–2019	^50^
Change in urbanization	2015–2019 average change in urban land cover at pixel level where household is located	MinMax Normalized	Wang, et. al., 2017	^51^
Distance to buildings	Distance of household to built infrastructure	MinMax Normalized	CIESIN, 2013	^52^
Distance to roads	Distance of household to built infrastructure	MinMax Normalized	CIESIN, 2013	^52^
Distance to PA	Distance of household to nearest Protected Area	MinMax Normalized	UNEP-WCMC, IUCN, 2022	^53^
Size of nearest PA	Spatial extent of nearest Protected Area	MinMax Normalized	UNEP-WCMC, IUCN, 2022	^53^
Standard deviation of NDVI	Standard deviation of NDVI for period 2015—2019	MinMax Normalized	Didan, K., 2021	[57]
Standard deviation of NPP	Standard deviation of NPP for period 2015—2019	MinMax Normalized	Didan, K., 2021	[57]
Threats	Level of anthropogenic threats to nature in pixel where household is located	MinMax Normalized	Bowler, et. al., 2020	^54^
Flooding	Level of exposure to flooding disasters in pixel where household is located	MinMax Normalized	Rosvold & Buhaug, 2021	^55^
Droughts	Level of exposure to drought disasters in pixel where household is located	MinMax Normalized	Rosvold & Buhaug, 2021	^55^
Temperature extreme	Level of exposure to extreme heat in pixel where household is located	MinMax Normalized	Rosvold & Buhaug, 2021	^55^
Educational attainment	Educational attainment of survey respondent	MinMax Normalized	Afrobarometer, q97	^23^
Perceived Security	Measure of whether household members feel safe in their location	MinMax Normalized	Afrobarometer, q10a	^23^
Physical Security	Measure of household members experiencing violence in their location	MinMax Normalized	Afrobarometer, q11b	^23^
Representative government	Measure of whether elected leaders represent interests of the people of their own	MinMax Normalized	Afrobarometer, q50	^23^
Freedom of Speech	Measure of freedom of speech	MinMax Normalized	Afrobarometer, q15a	^23^
Voting freedom	Measure of freedom of voting without coercion	MinMax Normalized	Afrobarometer, q15c	^23^

### Constructing random forest regression models under a quasi-experimental design

As discussed earlier, there is reason to believe that random forest machine learning models^39^ may be useful for examining the importance of factors in driving variation in observed wellbeing outcomes compared to the more traditional linear approaches commonly employed in the conservation literature^45^. This estimation technique relies on an ensemble learning approach that uses multiple decision trees to classify the outcome variable according to the influence of the variables. Each decision tree is used to predict the outcome in a separate model and the results of the ensemble are trained using a subset of the original data. The results of the ensemble of trees are then averaged to create the regression or prediction algorithm and are then applied to the entire dataset. This approach overcomes some of the limitations of classical linear models by relaxing the imposition of directionality and instead learning from the extant patterns in the dataset to identify the relative importance of each variable in driving movement in the response variable. Using a high number of simulation runs, the approach also minimizes the potential for decision trees to split based on unimportant regressors, thereby providing added confidence in variable importance scores^40^. This approach is limited in that its outcomes are not generalizable, as the model outputs cannot be extrapolated outside the existing data. However, given the contextual specificity of social-ecological relationships of households to PAs and natural resources around the world, we view this model as appropriate for illuminating the relative importance of variables, thereby enabling future site-based studies to unpack those relationships in detail.

To run the random forest models for the dataset, we utilized the ‘ranger’ package in R^41^, conducting predictions to measure accuracy and then running regression models for the full sample on three outcome variables described in Table 4: Overall wellbeing, Objective wellbeing, and Subjective wellbeing. We first constructed a training dataset using a subset of the data, and then ran a model using the full dataset. For the model, we set the number of simulations to 1000. We include all variables included in Table 5 to determine the importance scores of each. We analyzed the outputs by first comparing importance scores from model outputs, and later by constructing and examining ALE plots for each variable. The basic code for the random forest machine learning models is included in the supplemental material file.

Recent studies have found a relationship between household wellbeing using experimental designs with the treatment condition being located within 10 km from a PA and the control condition being located outside the 10 km buffer in multi-country studies across the developing world^18^. Others have found similar impacts using larger and smaller buffers in more constrained geographies, for instance, 5 km buffers^9^. We assume that variables including distance of a household to PA and size of nearest PA should have distinct relationships within such buffer zones compared to outside the buffer. Based on that assumption, we split the sample into those households that were within 10 km of a PA and those that were more than 10 km from a PA based on the design of^18^. To test whether we could utilize a quasi-experimental design to extend the study beyond the Random Forest approach alone, we matched households inside the 10 k buffer with households outside on a variety of the factors above. Rather than attempting propensity score matching given the need to discard unmatched observations, we reweighted the samples to balance the covariates using entropy balancing per previous studies^38^. In addition to the balancing procedure, country-level fixed effects were included in a linear model. However, the predictive power of the model was very low, and the assumption of linearity was unlikely. The inclusion of categorical variables and the likely non-linear relationship between those variables and wellbeing required a more flexible model therefore, we utilized these covariates in the machine-learning random forest regression model presented in this study, run on the two subsamples. While this is not the same experimental approach previous studies use, segregating the data according to the buffer and repeating our random forest model provides insight into the importance of absolute distance as a driver of movement in observed wellbeing scores within and outside of the buffer.

### Supplementary Information

Supplementary Information.

## Data Availability

The georeferenced household survey data are restricted and licensed by Afrobarometer. Data requests should be directed to them and request submission instructions are provided online at https://www.afrobarometer.org.

## References

[1] Bradshaw CJA, Ehrlich PR, Beattie A, Ceballos G, Crist E, Diamond J (2021). Underestimating the challenges of avoiding a ghastly future. Front. Conserv. Sci..

[2] Steffen W, Rockström J, Richardson K, Lenton TM, Folke C, Liverman D (2018). Trajectories of the earth system in the anthropocene. PNAS.

[3] Dinerstein E, Vynne C, Sala E, Joshi AR, Fernando S, Lovejoy TE, Mayorga J, Olson D, Asner GP, Baillie JE, Burgess ND (2019). A Global deal for nature: guiding principles, milestones, and targets. Sci. Adv..

[4] CBD (2022) Kunming-Montreal Global Biodiversity Framework. CBD/COP/15/L.25. Convention on Biological Diversity. Available online at: https://www.cbd.int/doc/c/e6d3/cd1d/daf663719a03902a9b116c34/cop-15-l-25-en.pdf. Last accessed on 5 January 2023.

[5] Maxwell SL, Cazalis V, Dudley N, Hoffmann M, Rodrigues AS, Stolton S, Visconti P, Woodley S, Kingston N, Lewis E, Maron M (2020). Area-based conservation in the twenty-first century. Nature.

[6] IPBES (2019). In E. S. Brondizio, J. Settele, S. Díaz, & H. T. Ngo (Eds.) Global assessment report on biodiversity and ecosystem services of the intergovernmental science-policy platform on biodiversity and ecosystem services. IPBES Secretariat.

[7] Hanson T, Brooks T, Da Fonseca G, Hoffmann M, Lamoreux J, Machlis G, Mittermeier C, Mittermeier R, Pilgrim J (2009). Warfare in Biodiversity Hotspots. Conserv. Biol..

[8] Fisher, J. (2022). Managing environmental conflict: an earth institute sustainability primer. New York. Columbia University Press. EISBN 978-0-231-55186-1

[9] Daskin JH, Pringle RM (2018). Warfare and wildlife declines in Africa’s protected areas. Nature.

[10] Golden RE, Kroner SQ, Cook CN, Krithivasan R, Pack SM, Bonilla OD, Cort-Kansinally KA, Coutinho B, Feng M, Garcia MIM, He Y, Kennedy CJ, Lebreton C, Ledezma JC, Lovejoy TE, Luther DA, Parmanand Y, Ruíz-Agudelo CA, Yerena E, Zambrano VM, Mascia MB (2019). The uncertain future of protected lands and waters. Science.

[11] Appleton MR, Courtiol A, Emerton L (2022). Protected area personnel and ranger numbers are insufficient to deliver global expectations. Nat. Sustain..

[12] Gatiso TT, Kulik L, Bachmann M, Bonn A, Bösch L, Freytag A, Heurich M, Wesche K, Winter M (2022). Sustainable protected areas: synergies between biodiversity conservation and socioeconomic development. People Nat..

[13] Fisher J, Allen S, Woomer A, Crawford A (2023). Protected area management and governance under pressure: an online survey to assess how to manage critical ecosystems for attainment of social and environmental goals and reduce stakeholder conflict. World Dev. Sustain..

[14] Betley EC, Sigouin A, Pascua PA, Cheng SH, MacDonald KI, Arengo F, Aumeeruddy-Thomas Y, Caillon S, Isaac ME, Jupiter SD, Mawyer A (2023). Assessing human well-being constructs with environmental and equity aspects: a review of the landscape. People Nat..

[15] Kruczkiewicz A (2021). Preparing for Compound risks and complex emergencies in a time of COVID. Proceed. Nat. Acad. Sci..

[16] Geldmann J, Joppa L, Burgess N (2014). Mapping change in human pressure globally on land and within protected areas. Conserv. Biol..

[17] McKinnon MC, Cheng SH, Dupre S, Edmond J, Garside R, Glew L, Woodhouse E (2016). What are the effects of nature conservation on human wellbeing? A systematic map of empirical evidence from developing countries. Environ. Evidence.

[18] Naidoo R, Gerkey D, Hole D, Pfaff A, Ellis AM, Golden CD, Fisher B (2019). Evaluating the impacts of protected areas on human wellbeing across the developing world. Sci. Adv..

[19] Ghoddousi A, Loos J, Kuemmerle T (2022). An outcome-oriented, social-ecological framework for assessing protected area effectiveness. BioScience.

[20] Corrigan C, Robinson J (2018). Global review of social indicators used in protected area management evaluation. Conserv. Lett..

[21] Jones N, McGinlay J, Dimitrakopoulos PG (2017). Improving social impact assessment of protected areas: a review of the literature and directions for future research. Environ. Impact Assessment Rev..

[22] Loveridge R, Sallu S, Presha I, Marshall A (2020). Measuring human wellbeing: a protocol for selecting local indicators. Environ. Sci. Policy.

[23] Afrobarometer Data, [Round 6, 2015]. All available countries utilized. Available at http://www.afrobarometer.org. Last accessed 22 September 2022.

[24] Fisher J, Arora P, Chen S, Rhee S, Blaine T, Simangan D (2021). Four propositions on integrated sustainability: toward a theoretical framework to understand the environment, peace, and sustainability nexus. Sustain. Sci..

[25] Breiman L (2001). Random forests. Machine Learn..

[26] Saha S, Saha M, Mukherjee K, Arabameri A, Ngo PT, Paul GC (2020). Predicting the deforestation probability using the binary logistic regression, random forest, ensemble rotational forest, REPTree: a case study at the Gumani River Basin, India. Sci. Total Environ..

[27] Saha N, Gosh T (2021). GIS-based spatial prediction of recreational trail susceptibility in protected area of Sikkim Himalaya using logistic regression, decision tree and random forest model. Ecol. Inform..

[28] Arabameri A, Pradhan B, Rezaeid K (2019). Gully erosion zonation mapping using integrated geographically weighted regression with certainty factor and random forest models in GIS. J. Environ. Manag..

[29] BenYishay, A., Rotberg, R., Wells, J., Lv, Z., Goodman, S., Kovacevic, L., Runfola, D. 2017. Geocoding Afrobarometer Rounds 1–6: Methodology & Data Quality. AidData. Available online at http://docs.aiddata.org/ad4/pdfs/geocodingafrobarometer.pdf.

[30] De Muro P, Mazziotta M, Pareto A (2011). Composite indices of development and poverty: an application to MDGs. Social Indicators Res..

[31] Dutta I, Nogales R, Yalonetzky G (2021). Endogenous weights and multidimensional poverty: a cautionary tale. J. Dev. Econ..

[32] Mazziotta M, Pareto A (2016). On a generalized non-compensatory composite index for measuring socio-economic phenomena. Social Indicators Res..

[33] UNEP-WCMC (2017). Global Database on Protected Area Management Effectiveness User Manual 1. UNEP-WCMC: Cambridge, UK. Available at: http://wcmc.io/GD-PAME_User_Manual_EN.

[34] UNEP-WCMC, IUCN (2022). Protected Planet: The World Database on Protected Areas (WDPA). https://www.protectedplanet.net/en

[35] Cumming GS, Allen CR (2017). Protected areas as social–ecological systems: perspectives from resilience and social–ecological systems theory. Ecol. Appl..

[36] Gorelick N, Hancher M, Dixon M, Ilyushchenko S, Thau D, Moore R (2017). Google earth engine: planetary-scale geospatial analysis for everyone. Remote Sens. Environ..

[37] Bowler DE, Bjorkman AD, Dornelas M, Myers-Smith IH, Navarro LM, Niamir A, Bates AE (2020). Mapping human pressures on biodiversity across the planet uncovers anthropogenic threat complexes. People Nat..

[38] Hainmueller J (2012). Entropy balancing for causal effects: a multivariate reweighting method to produce balanced samples in observational studies. Political Anal..

[39] Breiman L (2001). Random forests. Machine Learn..

[40] Grömping U (2009). Variable importance assessment in regression: linear regression versus random forest. Am. Stat..

[41] Wright MN, Ziegler A (2017). Ranger: a fast implementation of random forests for high dimensional data in C++ and R. J. Stat. Softw..

[42] Powlen KA (2023). Identifying socioeconomic and biophysical factors driving forest loss in protected areas. Conserv. Biol..

[43] Galvin KA, Beeton TA, Luizza MW (2018). African community-based conservation: a systematic review of social and ecological outcomes. Ecology Society.

[44] Hirons S, Matilda Collines C, Singh M (2022). Assessing variation in the effectiveness of IUCN protected area categorisation. What remotely sensed forest integrity and human modification reveals across the major tropical forest biomes. Ecol. Indicators.

[45] Oparina, E., Kaiser, C., and N. Gentile. (2022). Human wellbeing and machine learning. Preprint. https://arxiv.org/pdf/2206.00574.pdf 10.1038/s41598-024-84137-1 PMC11723942 39794488

[46] Coleman PT, Liebovitch LS, Fisher J (2019). Taking complex systems seriously: visualizing and modeling the dynamics of sustainable peace. Global Policy.

[47] Elvidge CD, Zhizhin M, Ghosh T, Hsu FC, Taneja J (2021). Annual time series of global VIIRS nighttime lights derived from monthly averages: 2012 to 2019. Remote Sens..

[48] Hansen MC, Potapov PV, Moore R, Hancher M, Turubanova SA, Tyukavina A, Thau D, Stehman SV, Goetz SJ, Loveland TR, Kommareddy A (2013). High-resolution global maps of 21st-century forest cover change. Science.

[49] Copernicus Sentinel data [2015 - 2020]. Retrieved from ASF DAAC [Octber 1 2021], processed by ESA.

[50] Wang, P., C. Huang, E. C. Brown de Colstoun, J. C. Tilton, and B. Tan. 2017. Global Human Built-up and Settlement Extent (HBASE) Dataset from Landsat. Palisades, New York: NASA Socioeconomic Data and Applications Center (SEDAC). 10.7927/H4DN434S. Accessed 10/1/2021.

[51] Center for International Earth Science Information Network - CIESIN - Columbia University, and Information Technology Outreach Services - ITOS - University of Georgia. 2013. Global Roads Open Access Data Set, Version 1 (gROADSv1). Palisades, New York: NASA Socioeconomic Data and Applications Center (SEDAC). 10.7927/H4VD6WCT.

[52] UNEP-WCMC, IUCN (2022). Protected Planet: The World Database on Protected Areas (WDPA). https://www.protectedplanet.net/en.

[53] Rosvold EL, Buhaug H (2021). GDIS, a global dataset of geocoded disaster locations. Sci. Data.

[54] World Bank, World Development Indicators. (2015). The world by income and region. Available at: https://datatopics.worldbank.org/world-development-indicators/the-world-by-income-and-region.html. Last accessed 29 September 2022

[55] Didan, K. (2021). *MODIS/Terra Vegetation Indices 16-Day L3 Global 250m SIN Grid V061* . NASA EOSDIS Land Processes Distributed Active Archive Center. Accessed 2023-09-28 from 10.5067/MODIS/MOD13Q1.061

[56] Esri. "Topographic" [basemap]. Scale Not Given. "World Topographic Map". February 19, 2012. http://www.arcgis.com/home/item.html?id=30e5fe3149c34df1ba922e6f5bbf808f. (Sep 20, 2023)

