# Supplementary material for: Assessing the influence of landscape conservation and protected areas on social wellbeing using random forest machine learning
Source: Sci Rep. 2024 May 18;14:11357. doi: 10.1038/s41598-024-61924-4 (PMC11102467; doi:10.1038/s41598-024-61924-4)
Supplement: Supplementary file 1 — Supplementary Information. [file 41598_2024_61924_MOESM1_ESM.docx]

**Assessing the Influence of Landscape Conservation and Protected Areas on Social Well-being Using Random Forest Machine Learning**

**Supplemental Materials**

**Basic R code for random forest models presented in the manuscript 1**

#note – in order to run the Random Forest models below, a dataset must be constructed that compiles Afrobarometer Wave 6 data into composite indices of Overall Wellbeing, Objective Wellbeing, and Subjective Wellbeing as described in Table 4 of the manuscript. Those data are available upon request from the source cited in the manuscript.

#note – once the composite indices have been constructed, geospatial analysis and processing must be conducted to append household observations from the Afrobarometer with data for spatial predictor variables, outlined in Table 5 of the mansuscript.

#note – variable names must be updated based on the geospatial analysis and processing referred to above. Working directories must be set to local directories.

**#Load all relevant libraries**

library(haven)

library(labelled)

library(tidyverse)

library(tidymodels)

library(usemodels)

library(ranger)

library(iml)

library(ale)

set.seed(123)

#Read in dataset (in the case below, we read in from a STATA file)#

ABfulldatasept29<- read_dta("…")

View(ABfulldatasept29)

**##Run random forest models for full dataset##**

index_AB <- sample(2,nrow(`ABfulldatasept29`), replace= TRUE, prob = c(0.7, 0.3))

train_AB <- ABfulldatasept29[index_AB==1,]

test_AB <- ABfulldatasept29[index_AB==2,]

**#examine predictions for Overall Wellbeing#**

ranger_ABdata_fullpred <- ranger(mmx_ABoverallWBsept27 ~ mmx_HHinfra + mmx_threat_int + q3recode_q3 + mmx_villagedevelop + mmx_cropschange + mmx_urbanchange + mmx_drought + mmx_ndviSD + mmx_ab_obj_eduattainment + mmx_lightschange + mmx_treeschange + mmx_nppSD + mmx_gis_area + incomegroup + mmx_near_dist + mmx_roads_dist + mmx_bldg_dist + mmx_ab_sbj_percsecurity + mmx_ab_obj_physicalsecurity + mmx_ab_votingfreedom +

mmx_ab_freedomspeech + mmx_ab_repgovt + mmx_extreme_temperature + mmx_flood, data = train_AB, mtry = 5, num.trees = 1000, write.forest = TRUE, replace = TRUE, importance = "impurity")

predict.ABfull <- predict(ranger_ABdata_fullpred, data = test_AB)

msem = mean((predict.ABfull$predictions - test_AB$mmx_ABoverallWBsept27)^2)

rmsem= sqrt(msem)

print(rmsem)

**#examine predictions for Objective Wellbeing#**

ranger_ABdata_OBJpred <- ranger(mmx_ABoverallWBsept27 ~ mmx_HHinfra + mmx_threat_int + q3recode_q3 + mmx_villagedevelop + mmx_cropschange + mmx_urbanchange + mmx_drought + mmx_ndviSD + mmx_ab_obj_eduattainment + mmx_lightschange + mmx_treeschange + mmx_nppSD + mmx_gis_area + incomegroup + mmx_near_dist + mmx_roads_dist + mmx_bldg_dist + mmx_ab_sbj_percsecurity + mmx_ab_obj_physicalsecurity + mmx_ab_votingfreedom +

mmx_ab_freedomspeech + mmx_ab_repgovt + mmx_extreme_temperature +

mmx_flood, data = train_AB, mtry = 5, num.trees = 1000, write.forest = TRUE, replace = TRUE, importance = "impurity")

predict.ABObj <- predict(ranger_ABdata_OBJpred, data = test_AB)

mseObj = mean((predict.ABObj$predictions - test_AB$mmx_AB_objsept27)^2)

rmseObj= sqrt(mseObj)

print(rmseObj)

**# examine predictions for Subjective Wellbeing#**

ranger_ABdata_SBJpred <- ranger(mmx_AB_sbjsept27 ~ mmx_HHinfra + mmx_threat_int + q3recode_q3 + mmx_villagedevelop + mmx_cropschange + mmx_urbanchange + mmx_drought + mmx_ndviSD + mmx_ab_obj_eduattainment + mmx_lightschange + mmx_treeschange + mmx_nppSD + mmx_gis_area + incomegroup + mmx_near_dist + mmx_roads_dist + mmx_bldg_dist + mmx_ab_sbj_percsecurity + mmx_ab_obj_physicalsecurity + mmx_ab_votingfreedom +

mmx_ab_freedomspeech + mmx_ab_repgovt + mmx_extreme_temperature + mmx_flood,

data = train_AB, mtry = 5, num.trees = 1000, write.forest = TRUE, replace = TRUE, importance = "impurity")

predict.ABSbj <- predict(ranger_ABdata_SBJpred, data = test_AB)

mseSbj = mean((predict.ABSbj$predictions - test_AB$mmx_AB_sbjsept27)^2)

rmseSbj= sqrt(mseSbj)

print(rmseSbj)

**# run ranger on full sample for Overall Wellbeing #**

ranger_ABdata_overall <- ranger(mmx_ABoverallWBsept27 ~ mmx_HHinfra + mmx_threat_int + q3recode_q3 + mmx_villagedevelop + mmx_ab_sbj_percsecurity + mmx_cropschange + mmx_urbanchange + mmx_drought + mmx_ndviSD + mmx_ab_obj_eduattainment +

mmx_lightschange + mmx_treeschange + mmx_nppSD + mmx_gis_area + incomegroup + mmx_near_dist + mmx_roads_dist + mmx_bldg_dist + mmx_ab_sbj_percsecurity + mmx_ab_obj_physicalsecurity + mmx_ab_votingfreedom + mmx_ab_freedomspeech + mmx_ab_repgovt + mmx_extreme_temperature + mmx_flood, data = ABfulldatasept29, mtry = 5, num.trees = 1000, write.forest = TRUE, replace = TRUE, importance = "impurity")

print(ranger_ABdata_overall)

importance(ranger_ABdata_overall)

**# run ranger on full sample for Objective Wellbeing #**

ranger_ABdata_Obj <- ranger(mmx_AB_objsept27 ~ mmx_HHinfra + mmx_threat_int + q3recode_q3 + mmx_villagedevelop + mmx_cropschange + mmx_urbanchange + mmx_drought + mmx_ndviSD + mmx_ab_obj_eduattainment + mmx_lightschange + mmx_treeschange + mmx_nppSD + mmx_gis_area + incomegroup + mmx_near_dist + mmx_roads_dist + mmx_bldg_dist + mmx_ab_sbj_percsecurity +mmx_ab_obj_physicalsecurity + mmx_ab_votingfreedom +

mmx_ab_freedomspeech + mmx_ab_repgovt + mmx_extreme_temperature +

mmx_flood, data = ABfulldatasept29, mtry = 5, num.trees = 1000, write.forest = TRUE, replace = TRUE, importance = "impurity")

print(ranger_ABdata_Obj)

importance(ranger_ABdata_Obj)

**# run ranger on full sample for Subjective Wellbeing #**

ranger_ABdata_Sbj <- ranger(mmx_AB_sbjsept27 ~ mmx_HHinfra + mmx_threat_int + q3recode_q3 + mmx_villagedevelop + mmx_cropschange +

mmx_urbanchange + mmx_drought + mmx_ndviSD + mmx_ab_obj_eduattainment +

mmx_lightschange + mmx_treeschange + mmx_nppSD + mmx_gis_area + incomegroup + mmx_near_dist + mmx_roads_dist + mmx_bldg_dist + mmx_ab_sbj_percsecurity +

mmx_ab_obj_physicalsecurity + mmx_ab_votingfreedom + mmx_ab_freedomspeech + mmx_ab_repgovt + mmx_extreme_temperature + mmx_flood,

data = ABfulldatasept29, mtry = 5, num.trees = 1000, write.forest = TRUE, replace = TRUE, importance = "impurity")

print(ranger_ABdata_Sbj)

importance(ranger_ABdata_Sbj)

**#note that once random forest models are estimated, ALE plots can be generated for all predictors**

**##Run random forest models for distance disaggregated dataset##**

**#Split sample within 10km from PA (here after called treatement) #**

trtAB<- subset(ABfulldatasept29, tenkmfromPA==1)

controlAB<- subset(ABfulldatasept29, tenkmfromPA==0)

index_trtAB <- sample(2,nrow(`trtAB`), replace= TRUE, prob = c(0.7, 0.3))

train_trtAB <- trtAB[index_trtAB==1,]

test_trtAB <- trtAB[index_trtAB==2,]

**#Split sample outside 10km from PA (hereafter called control)#**

index_ctrlAB <- sample(2,nrow(`controlAB`), replace= TRUE, prob = c(0.7, 0.3))

train_ctrlAB <- controlAB[index_ctrlAB==1,]

test_ctrlAB <- controlAB[index_ctrlAB==2,]

**#predictions of overall WB for treatment#**

ranger_ABdata_trtpred <- ranger(mmx_ABoverallWBsept27 ~ mmx_HHinfra + mmx_threat_int + q3recode_q3 + mmx_villagedevelop + mmx_cropschange + mmx_urbanchange + mmx_drought + mmx_ndviSD + mmx_ab_obj_eduattainment + mmx_lightschange + mmx_treeschange + mmx_nppSD + mmx_gis_area + incomegroup + mmx_near_dist + mmx_roads_dist + mmx_bldg_dist + mmx_ab_sbj_percsecurity + mmx_ab_obj_physicalsecurity + mmx_ab_votingfreedom +

mmx_ab_freedomspeech + mmx_ab_repgovt + mmx_extreme_temperature + mmx_flood,

data = train_trtAB, mtry = 5, num.trees = 1000, write.forest = TRUE, replace = TRUE, importance = "impurity")

predict.ABtrtOverall <- predict(ranger_ABdata_trtpred, data = test_trtAB)

msetrtOverall = mean((predict.ABtrtOverall$predictions - test_trtAB$mmx_ABoverallWBsept27)^2)

rmsetrtOverall= sqrt(msetrtOverall)

print(msetrtOverall)

**#predictions of overall WB for control#**

ranger_ABdata_ctrlpred <- ranger(mmx_ABoverallWBsept27 ~ mmx_HHinfra + mmx_threat_int + q3recode_q3 + mmx_villagedevelop + mmx_cropschange + mmx_urbanchange + mmx_drought + mmx_ndviSD + mmx_ab_obj_eduattainment + mmx_lightschange + mmx_treeschange + mmx_nppSD + mmx_gis_area + incomegroup + mmx_near_dist + mmx_roads_dist + mmx_bldg_dist + mmx_ab_sbj_percsecurity + mmx_ab_obj_physicalsecurity + mmx_ab_votingfreedom +

mmx_ab_freedomspeech + mmx_ab_repgovt + mmx_extreme_temperature + mmx_flood,

data = train_ctrlAB, mtry = 5, num.trees = 1000, write.forest = TRUE, replace = TRUE, importance = "impurity")

predict.ABctrl_Overall <- predict(ranger_ABdata_ctrlpred, data = test_ctrlAB)

mseCtrlOverall = mean((predict.ABctrl_Overall$predictions - test_ctrlAB$mmx_ABoverallWBsept27)^2)

rmseCtrlOverall = sqrt(mseCtrlOverall)

print(rmseCtrlOverall)

**#importance runs for OVERALL WB#**

**#treatment#**

ranger_ABdata_trt <- ranger(mmx_ABoverallWBsept27 ~ mmx_HHinfra + mmx_threat_int + q3recode_q3 + mmx_villagedevelop + mmx_cropschange +

mmx_urbanchange + mmx_drought + mmx_ndviSD + mmx_ab_obj_eduattainment +

mmx_lightschange + mmx_treeschange + mmx_nppSD + mmx_gis_area + incomegroup + mmx_near_dist + mmx_roads_dist + mmx_bldg_dist + mmx_ab_sbj_percsecurity +

mmx_ab_obj_physicalsecurity + mmx_ab_votingfreedom + mmx_ab_freedomspeech + mmx_ab_repgovt + mmx_extreme_temperature + mmx_flood,

data = trtAB, mtry = 5, num.trees = 1000, write.forest = TRUE, replace = TRUE, importance = "impurity")

print(ranger_ABdata_trt )

importance(ranger_ABdata_trt)

**#importance runs for OVERALL WB#**

**#control#**

ranger_ABdata_ctrl <- ranger(mmx_ABoverallWBsept27 ~ mmx_HHinfra + mmx_threat_int + q3recode_q3 + mmx_villagedevelop + mmx_cropschange + mmx_urbanchange + mmx_drought + mmx_ndviSD + mmx_ab_obj_eduattainment +

mmx_lightschange + mmx_treeschange + mmx_nppSD + mmx_gis_area + incomegroup + mmx_near_dist + mmx_roads_dist + mmx_bldg_dist + mmx_ab_sbj_percsecurity +

mmx_ab_obj_physicalsecurity + mmx_ab_votingfreedom + mmx_ab_freedomspeech + mmx_ab_repgovt + mmx_extreme_temperature + mmx_flood, data = controlAB, mtry = 5, num.trees = 1000, write.forest = TRUE, replace = TRUE, importance = "impurity")

print(ranger_ABdata_ctrl)

importance(ranger_ABdata_ctrl)

**ALE plots for all predictors included in Table 2 of the manuscript**

**
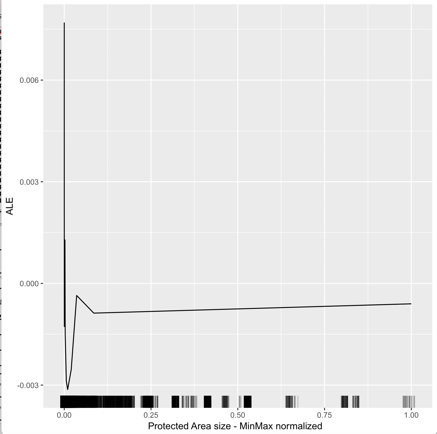
**

**
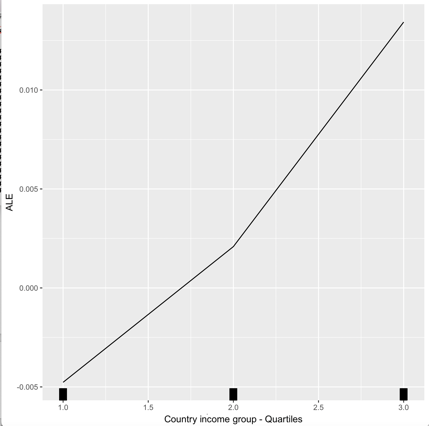

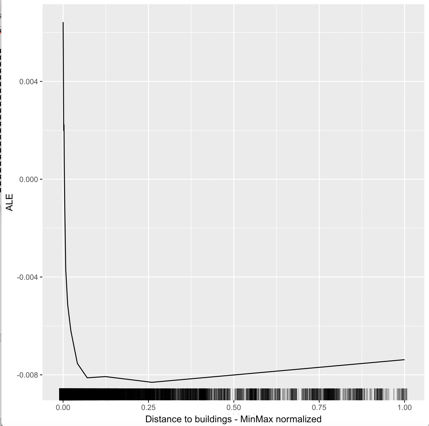
**

**
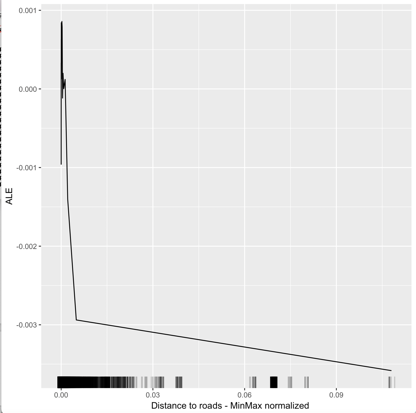

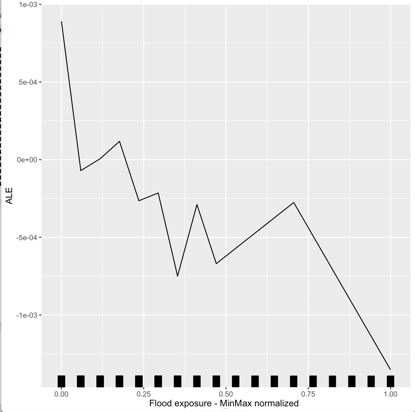

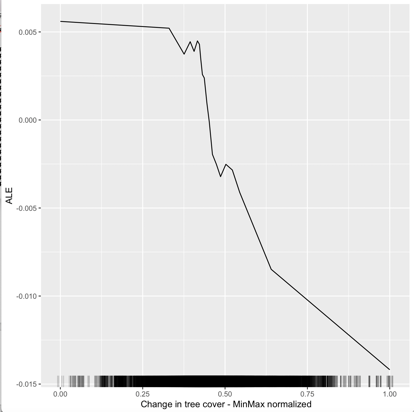
**

**
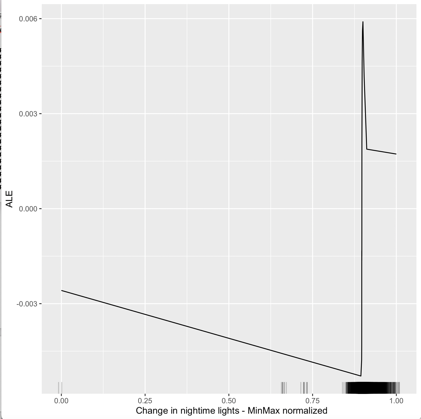

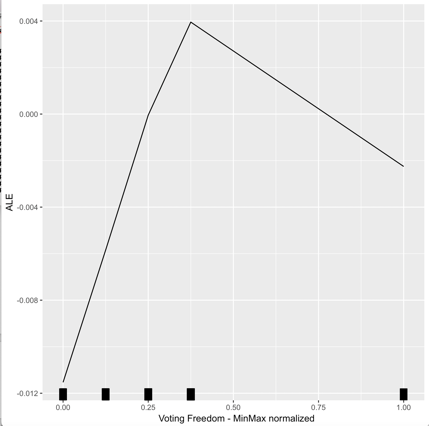

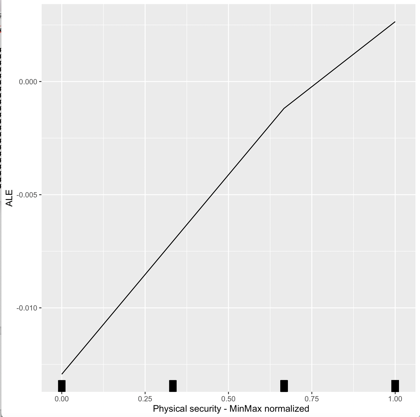
**

**
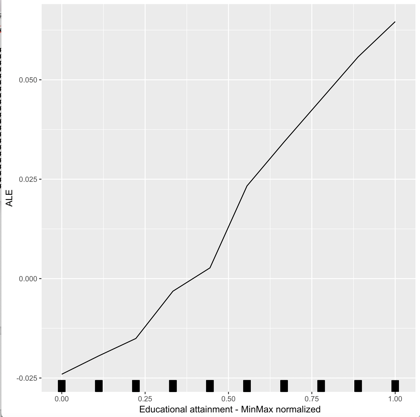

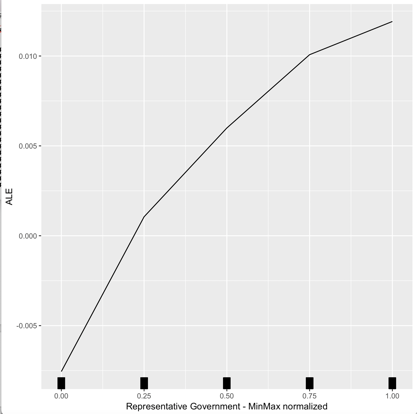

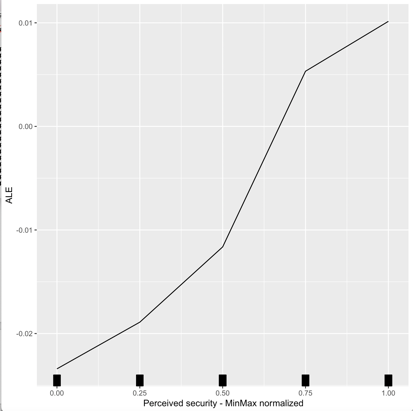
**

**
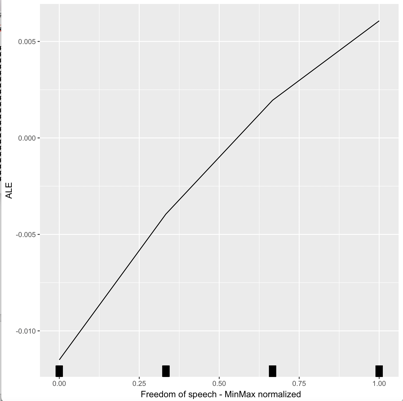

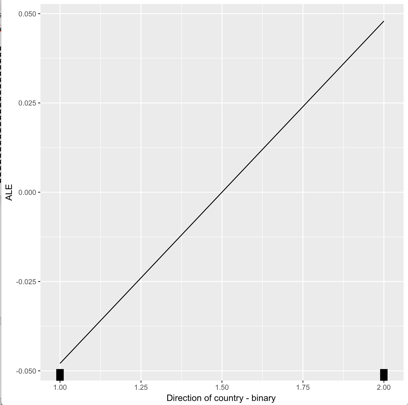

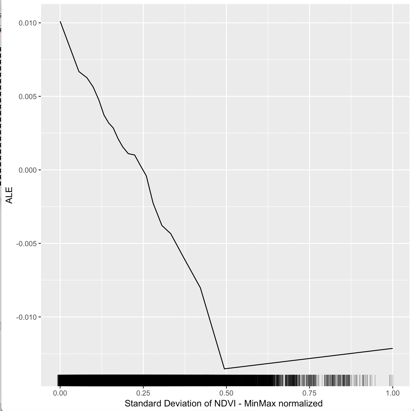
**

**
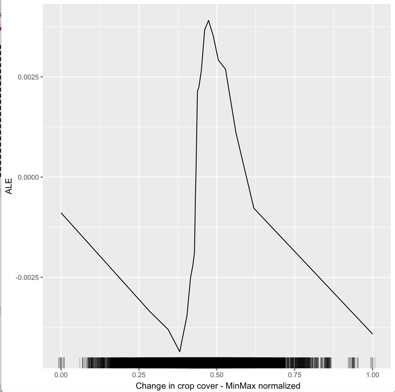

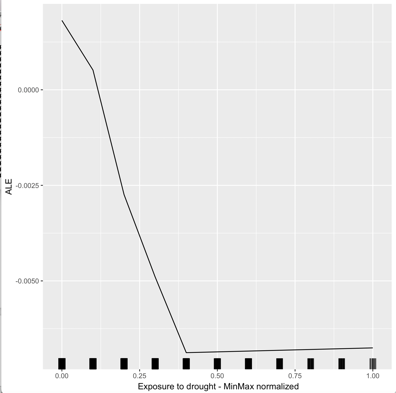
**

**
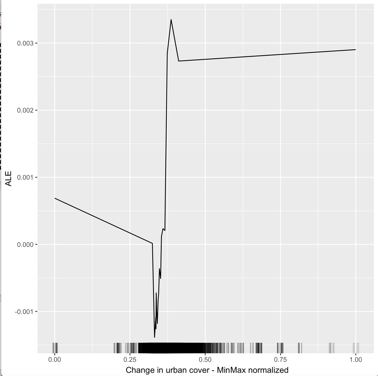
**

**
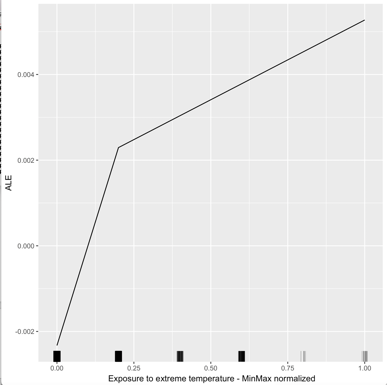

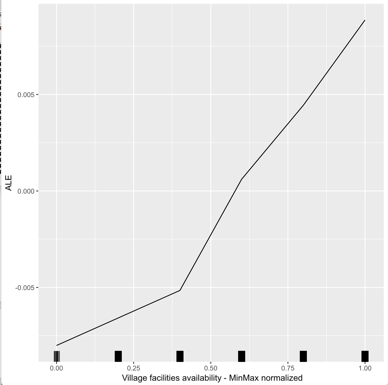

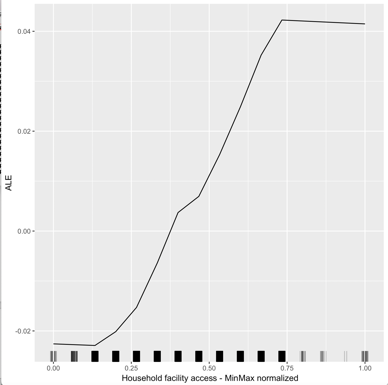
**

**
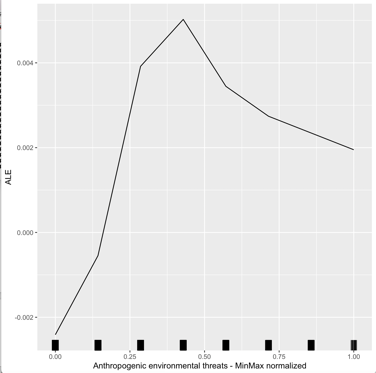

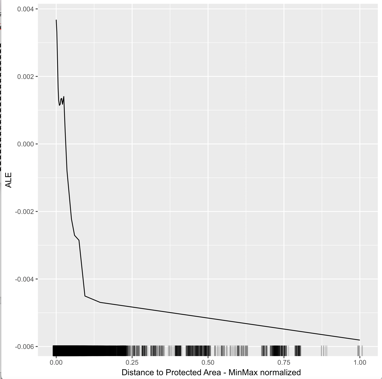
**
